# The role of NGOs in global health research for development

**DOI:** 10.1186/1478-4505-3-3

**Published:** 2005-02-21

**Authors:** Hélène Delisle, Janet Hatcher Roberts, Michelle Munro, Lori Jones, Theresa W Gyorkos

**Affiliations:** 1 Département de nutrition, Faculté de médecine, Université de Montréal, C.P. 6128 succ. Centre-ville, Montréal, Qc, H3C 3J7, Canada; 2 Canadian Society for International Health (CSIH), One Nicholas Street, Suite 1105, Ottawa, On K1N 7B7, Canada; 3 CARE Canada Suite 200 9 Gurdwara Road Ottawa, ON, K2E7X6 Canada; 4 Canadian Society for International Health (CSIH), One Nicholas Street, Suite 1105, Ottawa, On K1N 7B7, Canada; 5 Dept. of Epidemiology & Biostatistics, Montreal General Hospital, 1650 Cedar Avenue, Room L-10-420, Montreal, QC H3G 1A4, Canada

## Abstract

**Background:**

Global health research is essential for development. A major issue is the inequitable distribution of research efforts and funds directed towards populations suffering the world's greatest health problems. This imbalance is fostering major attempts at redirecting research to the health problems of low and middle income countries. Following the creation of the Coalition for Global Health Research – Canada (CGHRC) in 2001, the Canadian Society for International Health (CSIH) decided to review the role of non-governmental organizations (NGOs) in global health research. This paper highlights some of the prevalent thinking and is intended to encourage new thinking on how NGOs can further this role.

**Approach:**

This paper was prepared by members of the Research Committee of the CSIH, with input from other members of the Society. Persons working in various international NGOs participated in individual interviews or group discussions on their involvement in different types of research activities. Case studies illustrate the roles of NGOs in global health research, their perceived strengths and weaknesses, and the constraints and opportunities to build capacity and develop partnerships for research.

**Highlights:**

NGOs are contributing at all stages of the research cycle, fostering the relevance and effectiveness of the research, priority setting, and knowledge translation to action. They have a key role in stewardship (promoting and advocating for relevant global health research), resource mobilization for research, the generation, utilization and management of knowledge, and capacity development. Yet, typically, the involvement of NGOs in research is downstream from knowledge production and it usually takes the form of a partnership with universities or dedicated research agencies.

**Conclusion:**

There is a need to more effectively include NGOs in all aspects of health research in order to maximize the potential benefits of research. NGOs, moreover, can and should play an instrumental role in coalitions for global health research, such as the CGHRC. With a renewed sense of purpose and a common goal, NGOs and their partners intend to make strong and lasting inroads into reducing the disease burden of the world's most affected populations through effective research action.

## 

"*Each country needs to be able to generate knowledge relevant to its own situation, to allow it to determine its particular health problems, appraise the measures available for dealing with them, and choose the actions likely to produce the greatest improvement in health. This should not be seen as the exclusive preserve of universities or research councils, but equally of health/public services, non-governmental organizations, etc." *[1].

## 1 Introduction

Non-governmental organizations (NGOs) have been defined by the World Bank as 'private organizations that pursue activities to relieve suffering, promote the interests of the poor, protect the environment, provide basic social services, or undertake community development'. NGO activities can be local, national or international. NGOs have contributed to the development of communities around the world and are important partners of many governments – while remaining independent from governments. According to the Human Development Report [2], there were in 2002 over 37,000 NGOs in the world, a growth of 19.3% from 1990. Their purposes differ but overall two categories dominate: economic development and infrastructure (26%) and research (23%) . NGOs are generally regarded as valued partners in health research for development, research being viewed as a broad process involving not only the production of knowledge, but also up-stream and down-stream activities needed for its relevance and effectiveness, such as priority setting and knowledge translation. NGOs have made and continue to make substantive contributions through supporting relevant and effective research. In her address at the First Steering Committee Meeting of the International Conference on Health Research for Development in 1999, the (then) Director General of the World Health Organization (WHO), Dr. Gro Harlem Brundtland, voiced her appreciation of NGOs as a partner with WHO in health research [3].

There are several views on what is meant by global health and global health research. In its simplest form, global health is population health on a global scale, and global health research is research which addresses the health of human populations around the globe. Global health also refers to 'inherently global health issues', that is, health-determining phenomena that transcend national borders and political jurisdictions, such as globalization and climate change. In setting global health research priorities, both the burden of disease and inherently global issues should be considered [4,5]. The vision of health research as proposed by the Commission on Health Research for Development [6] is a systems approach driven by equity, focused on country needs and priorities, and within an interactive regional and global framework. This paper will address global health as it was defined in a Canadian consultation paper on global health research held in 2001 , that is, the health of individuals and societies in less developed, less resourced, poorer nations and regions of the world.

A major global health research issue is the inequitable distribution of research efforts and funds directed towards populations suffering the world's greatest health problems. This situation has been referred to as the 10/90 gap because only a meager 10% of all health research funding is being used to address 90% of the world's burden of disease, suffered primarily in developing countries [7]. Because of this imbalance, there have been major attempts at redirecting research efforts and funds to the health problems of low and middle income countries.

One of the roles of health research is to ensure that the measures proposed to break out of the vicious cycle of ill health and poverty are based, as far as possible, on evidence, so that the resources available to finance these measures are used in the most efficient and effective way possible [8]. There are many different types of health research. At the 6^th ^Global Forum on Health Research, held in Arusha, Tanzania in November 2002, Dr. Gerald Keusch, Director of the Fogarty International Center, listed the scope of health research as including: fundamental discovery research, pathogenesis research, epidemiology research, clinical research, product development research, translational and adaptational research, operational research, health services research, policy research and research on health systems [9]. NGOs involved in health research have primarily undertaken operational and action research, but many have also participated in other types of research such as epidemiological research, social science research, product development research, translational research, health services research, and policy research.

The purpose of this paper is to document the role that NGOs have played in global health research and to highlight the need to expand this role. This paper is also intended as a tool to stimulate research activity in NGOs and to advocate for increased NGO involvement in global health research. Following a brief review on the central role of global health research in development, the roles of NGOs at different stages within the research process are discussed and illustrated with a few examples. Key challenges are also identified. The last part of the paper identifies future needs for strengthening the role of NGOs in global health research.

## 2 Global Health Research and Development

**Figure 1:**
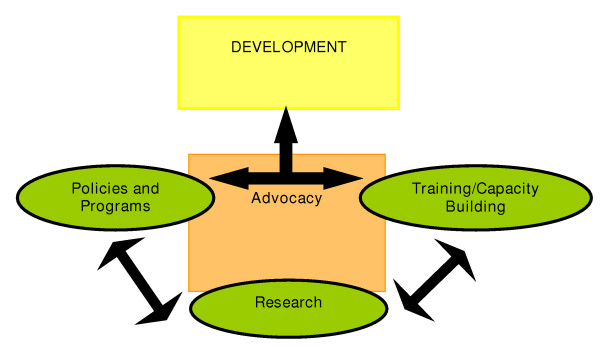
The relationship between research and development

While research means different things to different people, it may best be defined as 'a knowledge loop' from generation of knowledge to its effective use [10]. Indeed, there has been a progressive paradigm shift from narrow 'research' to broader 'knowledge creation and management' [11]. This broad definition is consistent with that of the Organization for Economic Co-operation and Development (OECD) [12] which states that "research and experimental development comprise creative work undertaken on a systematic basis in order to increase the stock of knowledge, including knowledge of man, culture and society, and the use of this stock of knowledge to devise new application". Research is recognized as a fundamental ingredient for action [13,14], and it is essential for development because it informs policies and programs; it also guides the development of human resources in these and related domains (see Figure 1). However, the links among research, policy-making, programming and training, with advocacy constantly in the background, need to be strengthened. It is being increasingly recognized that investments in health research can be economic and social investments [15]. In a WHO discussion paper on knowledge for better health, the emphasis is on research as an investment rather than a cost, on the need to turn research into action, and on the vital part of the civil society ( World report on knowledge for better health 2004).

### 2.1 Global health research priorities

The call to shift health research priorities from problems of industrialized countries to those affecting populations in developing countries is not new. In 1990, concerns regarding the inequitable distribution of research efforts were first raised in the Report to the Commission on Health Research for Development [6]. Since then, progress has been made to try to correct this gap, and to build capacity in the countries of greatest need. The 2002 WHO World Health Report [16] focuses on risks that contribute to the global burden of disease and death, both in developing and developed countries. Dollar expenditures on health research today, however, remain markedly inequitable in terms of populations served and disease burden addressed. Pneumonia, diarrheal diseases, tuberculosis and malaria, when combined, have been estimated to account for more than 20% of the disease burden in the world (mostly in developing countries), yet they receive less than 1% of the total public and private funds which are devoted to health research. The 10/90 gap is as wide as ever [7].

### 2.2 Milestones in global health research and development

Several important initiatives have been undertaken to address the global health research agenda. They have been fostered by individuals and groups from local, national and international bodies who shared a common vision in advocating for health research directed towards the low and middle income countries.

#### 2.2.1 Commission on Health Research for Development

The Commission on Health Research for Development declared in 1990 that "For the most vulnerable people, the benefits of research offer a potential for change that has gone largely untapped" [6]. The Commission highlighted several obstacles in undertaking this research, and among others: 1) the insufficient (worldwide) funding of health research directed towards health problems of people in developing countries; 2) the inefficient application of resources; 3) the neglect of major health problems; 4) the lack of individual and institutional health research capacity; 5) the lack of technology transfer; and 6) fragmentation and competition among research initiatives. The challenge to remedy this situation was set down and ultimately led to the establishment of the Council for Health Research in Development (COHRED) in 1993. COHRED works in partnership with WHO, the World Bank and other organizations to strengthen the role of health research at the country level.

Over the years, COHRED has assisted increasing numbers of countries in the exploration and implementation of essential national health research (ENHR) strategies. Networks were created to facilitate national level activities in Africa, Asia, and the Commonwealth Caribbean. For example, AFRO-NETS, the 'African Networks for Health Research and Development', was established in 1997 to facilitate exchange of information among different networks active in this type of research in English-speaking Africa, and to facilitate collaboration in the fields of capacity building, planning and research. Regional and global working groups and projects were established which allowed experiences with ENHR to be shared. Several communication strategies were utilized, including quarterly newsletters, websites and other publications to share experiences and lessons learned. A framework for capacity development, a critical component of ENHR, was established through partnerships and like-minded networks and organizations. The book, '*Forging Links for Health: Perspectives from the Council on Health Research for Development*", [14] and the discussion paper '*Health Research for Development: The Continuing Challenge *[1] review what has happened in the intervening years since the Commission on Health Research for Development made its first major recommendations in 1990.

Several questions remain unanswered:

• To what extent have the recommendations been implemented?

• Have the recommendations made a real difference in the lives of the countries that carry 90% of the disease burden?

• Has 'Essential National Health Research' worked?

• What is the current situation with regard to health research for development?

• Where and how do we proceed from here?

The 2000 International Conference on Health Research for Development provided COHRED and several partner organizations with an opportunity to review and reflect on their experience with health research, its impact on health and equity and to devise a global strategy for the first years of the coming millennium [14].

#### 2.2.2 Global Forum for Health Research

The Global Forum for Health Research, created in 1998 as a response to the Report of the WHO *ad hoc *Committee on Health Research Relating to Future Intervention Options [17], has provided a forum for stakeholders to review global health research priorities, promote ongoing analysis of the international health research situation and facilitate coalition building to support its central objective to help correct the 10/90 gap. The Global Forum is managed by a council of 20 members representing government policymakers, multilateral and bilateral agencies, foundations, international NGOs, women's associations, research institutions, and the private sector. It holds funding competitions on targeted global health topics and awards research grants to applicants from low and middle income countries. Its most recent report [18] emphasized the need for action by combined efforts of the public and private sectors. It also recognized the role of NGOs as a partner in contributing to these efforts.

#### 2.2.3 Canadian Coalition for Global Health Research

In November 2001, four Canadian federal agencies, Canadian International Development Agency (CIDA), International Development Research Centre (IDRC), Health Canada, and Canadian Institutes of Health Research (CIHR) signed a Memorandum of Understanding to support national consultation regarding Canada's role in global health research. This marked the first time in Canadian history that Canada's two overseas development agencies, Health Canada and Canada's major federal health research funding agency have collaborated to address global health research.

The Canadian Coalition for Global Health Research (CCGHR) is developing into a network of health researchers, funding agencies, NGOs, and other stakeholders committed to support the pursuit of effective global health research by ensuring that all these groups work together as effectively as possible with researchers in developing countries. This collaborative approach serves as a framework for future research projects in the area of global health, with each organization bringing its own specific area of expertise to the table. It aims to improve the effectiveness of development assistance and to increase the sustainable health gains per dollar of Canadian funds invested in research.

## 3 Key Roles of NGOs in Global Health Research

Inequities in health are caused by a number of determinants, including the use of or access to health care facilities. Research which addresses these issues requires an intersectoral approach, involving trans-disciplinary teams and methodologies. Building trans-disciplinary teams requires commitment from the research community to seek out colleagues from other disciplines, from the funding agencies to appreciate innovative initiatives, from the community at large as partners and contributors, and from the policy arena to develop strategies for intersectoral policies and programs which may well have the lead outside ministries of health. Indeed, working outside government altogether may well be a solid and sustainable strategy. Understanding and engaging the broader community on these issues comes naturally to communities unrestricted by bureaucratic boundaries. This is where NGOs excel.

**Figure 2:**
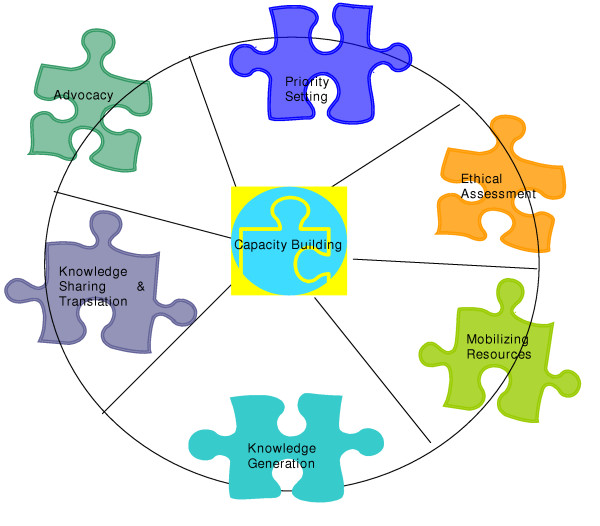
Research Process

NGOs have contributed to all different stages of the research cycle (see Figure 2), namely in advocacy, priority setting, capacity building, resource mobilization, sharing and utilization of research findings, and networking. Traditionally, many NGOs which have undertaken activities that address health issues in resource-poor settings are service-oriented NGOs and concentrate their efforts on implementing "action" programs. This type of NGO finds it difficult to identify resources that would allow them to conduct research. While there are NGOs involved in actually conducting research, for most the focus is usually evaluation. Links with the research community are often weak. Other NGOs undertake innovative field-based experimental research. The effectiveness of these initiatives is often learned by trial and error. Unfortunately, while this enhances effective and efficient implementation in the field, research results are only infrequently analyzed appropriately. There are also barriers to dissemination or sharing of research results to a wider audience (eg. other districts within the same country) and to different audiences (eg. to other researchers, research institutions, etc.). Typically, NGO involvement in research is more downstream of knowledge production and it usually takes the form of a partnership with more traditionally-oriented research organizations such as universities or dedicated research agencies. There is a need to include NGOs in the reconceptualization of global health research to ensure completion of the cycle from generation of knowledge to its effective use.

We describe the key roles of NGOs below, using, as a framework, the categories of primary functions of health research systems as recently identified by Butler [1].

### 3.1 Stewardship

One of the strengths of NGOs has been as advocates for the populations they serve. Health research can make NGOs become more effective advocates. Governments depend on health research for needs assessments, formulation of policy options, implementation of interventions and evaluation of action plans. Empowered citizens and NGOs can demand accountability of the government. They can also encourage international donors to focus on the health priorities of countries and thus facilitate a check and balance mechanism for good governance. Good governance is needed to improve collaboration and cooperation at the international, national and regional levels in order to tackle inequity. High scientific standards are fundamental components of effective health governance, particularly as they relate to health research systems.

The role of research in mobilizing and supporting NGOs, particularly around issues of inequities, is important. NGOs can provide stewardship in terms of the promotion and advocacy for relevant research, shaping research priorities, and the setting and interpretation of ethical frameworks for research. NGOs can often play a more powerful role using the results of research than can the research community itself. Mobilizing communities, utilizing mechanisms for advocacy and acting as an interface between the research community and its wider community will enhance a sense of strong governance and stewardship.

#### 3.1.1 Promotion and advocacy for relevant global health research

There is widespread agreement that health research is not sufficiently valued by many societies as a critical input to human and socioeconomic development. The result is often an environment that is neither conducive to, nor supportive of, research. A culture is necessary that recognizes the value of research and one which builds a supportive environment for research [19].

There is a need not just to allocate funds for research, but also to allocate these funds to areas of research that would have the greatest or maximum social benefit. Advocacy for relevant research, that is, the type of research that will make a difference in terms of equity, health, well-being and development of people, is an important role for NGOs [20]. Not only can NGOs identify researchable topics, but they can also stimulate demand for relevant research.

However, the existing power structure in the research arena often works against NGOs because of a narrow view of research as merely producing new knowledge, with limited consideration of upstream operations (identification of research needs, questions, and priorities), downstream actions (knowledge management, dissemination and translation), and the advocacy efforts required to connect research with policies, programs and training.

Historically, the influence of the biomedical researchers' lobby has been the strongest with regard to agenda-setting and fundraising. Behavioral scientists and social health researchers generally have much weaker potential to influence resource allocation, agenda-setting and policy formulation. Partnerships could be strengthened and supported between NGOs and social science researchers in resource-poor countries to improve influence potential, as the social sector issues that tend to be most relevant to human populations are also of utmost importance to NGOs.

Creating a favorable environment for "relevant" research requires a health system that is supportive and provides financing opportunities. It also requires the existence of a culture of "evidence-generating and evidence-based research". There must be a healthy relationship between communities, researchers and policy makers. Networks to share experiences, lessons learned and policy impact can be enhanced by partnerships with NGOs.

A disproportionately large number of people living in developing countries suffer large disease burdens. Promoting research and development on neglected diseases or issues of global health significance may contribute to bridging the 90/10 research gap, by stimulating research by public or civil society organizations on issues that do not represent marketable research, and are therefore neglected by the private sector. There is a role for NGOs in advocating for more research on these neglected topics (see under 4.1, example of initiative for neglected disease drugs, Médecins sans Frontières [MSF]).

Health research needs to generate knowledge that will facilitate the identification of choices and options to reinforce equity-based policies and programs. In doing so, it also needs to address the difficulties of collecting data that are of primary importance when inequities are discussed. The essential function that data serve will allow tracking and monitoring of resources for research and for improving opportunities for those researchers in more disadvantaged countries. NGOs often have access to information that will highlight inequities and the determinants of inequities.

Similarly, NGOs can advocate for formative and evaluative research on programs that address major health problems, but which are generally a low priority for funding agencies. In doing so, they can contribute to making data available for evidence-based decision-making in policy and program planning. Food system-based approaches to reducing micronutrient deficiencies and malnutrition in general are one of these under-researched areas.

#### 3.1.2 Shaping research priorities

NGOs are well-placed to foster public participation in decisions about health research, as they are close to communities. They can provide the mechanisms by which such public participation is ensured in decision-making processes. Significant progress has been made over the last decade in health research priority-setting for the implementation of ENHR at the country level. Among the lessons learned, it appears that community involvement is in most cases an unresolved issue [21]. What is certain is that, critically at the priority-setting stage of the research cycle, the community must be involved, and NGOs may be instrumental in achieving this.

Defining the research that needs to be done requires the input of civil society and NGOs as much at the beginning as at the end, in terms of dissemination, communication and action.

#### 3.1.3 Setting and interpreting ethical frameworks

NGOs assume a range of roles in research, but a thread that runs through all these is their representation and advocacy for the vulnerable. Broad research roles are described in greater detail in other sections of this paper. This section focuses on the role of NGOs in shaping and interpreting ethical frameworks [22-25], that is, the incorporation of ethical principles in their research partnerships with other organizations. As researchers or research partners, NGOs have a responsibility to ensure that ethical issues are addressed in both the design and conduct of the research. There are distinctive challenges in conducting health research in developing countries, namely to fulfill moral duties of justice and respect in the face of poverty, lack of resources and the potential for exploitation. The Nuffield Council on Bioethics [26] designed an ethical framework for health research in developing countries based on the duty to alleviate suffering, to show respect for persons, to be sensitive to cultural differences, and to not exploit the vulnerable. As NGO research is often conducted among the most vulnerable populations, where power relations are tipped in favor of researchers and those who are literate and eloquent, issues of informed consent and participants' understanding of it and the research, as well as participants having access to the benefits of research, are of special concern. Particularly when research is conducted by first world researchers in resource-limited settings, NGOs who partner in this research at times need to recommend and advocate for reviews from local research and ethics committees, as well as those from industrialized countries. Where relevant, they may also encourage the development of independent national ethics committees and national ethical guidelines, taking account of existing international guidelines [22-25]. This process may involve interpreting cultural ethical frameworks and beliefs, for instance, culturally appropriate means of obtaining informed consent from research participants. In addition, NGOs can make sure that the development of local expertise in health research is an integral component of research proposals.

As watchdogs, NGOs actively seek breaches of ethics and hold researchers to account when the principles of respect for persons, beneficence and justice are not upheld, a role they are well positioned to assume given their understanding of and links to marginalized groups. Watchdogs, as they uncover ethical breaches that may be defined by culture or power relations, have assisted in shaping ethical frameworks to better address ethics when research is conducted among vulnerable groups.

In the communities where NGOs work, they can act as community partner members of and witnesses to research. In this role they can assist with, for example, interpreting research objectives to participants to ensure that consent is informed and the rights of subjects are respected. They may provide researchers with enumerators or local information to expedite the data collection process. NGOs can also monitor the long-term outcomes arising from research, and make sure that the participants benefit from successful intervention.

As knowledge translators, NGOs interpret the knowledge generated by research to their constituents, a key role in working towards the vulnerable having access to the benefits of research that could improve their lives. This may be research conducted in these communities or globally.

### 3.2 Mobilizing resources for research

While current levels of financial resources are not sufficient to adequately respond to the demonstrated need for health research, there are many sources of "funds" for health research. Some are monetary contributions and some are in-kind contributions. NGOs can provide not only direct funding for projects (albeit in a limited manner) but, and perhaps equally important, they can provide valuable in-kind funding. Thus, personnel or materials developed by NGOs can be used in health research projects at little or no cost.

Some NGOs are directly involved in the administration of research grants. Others may be the fiduciary agent for a grant to a research organization that is exploring an issue related to an NGO program. However, most are organizations that work with communities. A major role is therefore to identify resource gaps using networks to link communities, health providers and managers, and funding agencies in a meaningful way so that financing can appropriately be directed to targeted health issues. NGOs may also contribute by identifying other potential sources of funding, for instance, in the local private sector.

### 3.3 Knowledge generation

Knowledge can be acquired in various ways, by many methods, and by different types of people; there are different cultures of enquiry. Because of their typical 'grass-roots' experience, several NGOs are able to access indigenous knowledge and specific information, which may be less attainable for other types of organizations. This type of knowledge might be very useful when pooled with knowledge acquired by others; in this way, a more comprehensive analysis can occur. NGOs can be particularly adept in conducting formative research (baseline studies, needs assessment), in operational or action research and in process and impact evaluation. This type of research is particularly relevant for setting priorities, for informing intervention, as well as for identifying further research needs.

Although knowledge generation is generally not a primary NGO activity, there may be specific 'knowledge generation' research niches for NGOs. For instance, as suggested by the Canadian Council for International Cooperation (CCIC) and actually carried out by a few NGOs, "*There is a need for NGOs to be more involved in policy research even in Canada*" (Interview with B. Tomlinson, CCIC).

**Figure 3:**
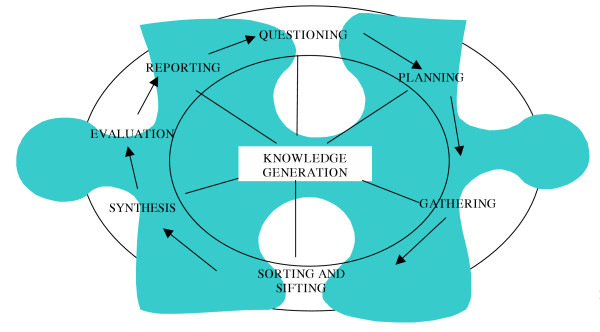
The research (knowledge generation) cycle (adapted from McKenzie [36])

Figure 3 illustrates the research cycle in the narrow sense of knowledge generation. This cycle applies whatever the research type, and whether the research is conducted by an NGO or an academic institution.

### 3.4 Utilization and management of knowledge

While asserting that the production of knowledge is the primary function of research, and that levels of knowledge have increased considerably, a discussion paper for the International Conference on Health Research [1] also recognizes that the ability to draw from research in terms of lessons learned, application to interventions, and programming and policies which support the overarching goal of equity, is often lacking.

Inadequacies include the inability of developing countries to access pertinent international research literature and knowledge bases (either as contributors or users), the inability to access new information technologies, and the inability to ensure closer links among the research community, health service managers and health policy makers.

The effective use of research findings and their dissemination is an increasingly important public health policy concern. In 1995, an international research conference was held in Vancouver, Canada, on dissemination research. This type of research is similar to what is now called 'translational research' , that is, the conversion of research findings from basic, clinical or epidemiological environmental health science research into information, resources, or tools that can be applied by health care providers and community residents to improve public health outcomes in at-risk neighborhoods.

NGOs are frequently at the interface of applied research and policy-making, at least at the administrative level, and their potential input into research utilization for policy-making needs to be valued. Research can make a substantive contribution in at least three phases of the policy-making process: agenda-setting, policy formulation, and implementation [27]. It is widely recognized that health research is underutilized in policy-making. The generation of new knowledge is highly valued, but its translation and use does not appear to be valued as much [28], which may partly explain why application of newer knowledge is often a weak link in the research cycle. Factors potentially enhancing utilization can be identified by exploration of priority-setting, activities of the health system at the interface between research and policy-making, and the role of recipients, or "receptors", of health research [27]. There are several models of research utilization in policy-making, but interactive or exchange models may be more conducive to the effective use of research than unilateral models because they bring researchers and decision-makers closer together [10,27].

NGOs often play a critical role in interpreting the evidence and translating its relevance for local communities. Inevitably the level of involvement by the community depends on relevance and opportunity for action and advocacy. Assessing and evaluating opportunities for advocacy and action occur as NGOs work with communities on these issues. Effective involvement of the community and its participation is a "matter of reciprocity and continuing dialogue in which participation takes different forms and influences change in several directions" [14]. Once the evidence has been analyzed and assimilated, NGOs can serve as intermediaries in delivering feedback to communities and in the planning, implementing and monitoring of new interventions, policies or other actions which might have been proposed. The knowledge and information acquired by NGOs can be unique and offer added insight into new ideas for future health research. This is, in part, because of the extensive interrelationships NGOs have forged with different communities, organizations, the private sector and governments, among others, often over decades of dedicated work. Additionally, NGOs are in a good position to test the ability of research findings to be scaled up in a 'real world' environment.

According to Lavis *et al *[10], while the "knowledge loop" needs to be completed, that is, from knowledge production to knowledge-based decision-making through knowledge transfer or brokering, not all research organizations should become involved in knowledge transfer; if they do, the knowledge pyramid may be shaky. Innovations stemming from research are at the base of the pyramid, and actionable messages are at the top. Individual studies and synthesis of research knowledge are the intermediate layers. Lavis *et al *contend that it may not be relevant to transfer knowledge from individual studies, but rather, from bodies of cumulative research knowledge, and that knowledge transfer brokers are needed for this purpose. This model of specialized roles is probably more relevant at the macro level and in industrialized countries. In resource-poor countries, polyvalent organizations such as NGOs have a key role in sharing, translating and implementing research findings at the community and country level. They provide channels for the use of research results at the community level, as they are closest to the communities themselves. For that very reason, they may also feel more compelled to complete the research cycle, including application of the findings. *Third World Network *, for instance, an independent non-profit international network of organizations and individuals involved in issues relating to development, conducts and disseminates research to help organizations around the world participate in and influence international economic and social policy. NGOs may also be involved in testing pilot models of intervention and in their subsequent scaling-up.

### 3.5 Capacity development

The preliminary examination of the functions performed by the some 125 organizations involved in a significant way in health research reveals that while knowledge generation is a concern shared by most, research capacity strengthening receives relatively little attention [1]. One weakness or inattention in research capacity strengthening activities, for example, has been the lack of a recognized career path for local health researchers which has resulted in diverting promising researchers to other careers or to other countries.

The development and retention of research capacity remains a challenge in many countries [29]. Quality control and assurance requires skills and structures which support these objectives. Skills such as leadership, advocacy, networking and communication are important and need to be built through capacity development. Research management is also a skill which needs to be strengthened and a skill that will improve the quality, appropriateness and timeliness of research and its dissemination.

NGOs in the North and in resource-poor countries often have the capacity for facilitating training and for sharing the lessons learned in needed skills. Partnership with NGOs in such capacity-building needs to be valued and reinforced. The Canadian Society for International Health (CSIH) and the Canadian Public Health Association (CPHA) have participated in capacity-building activities in many countries and continue to share their experiences and lessons learned. Support for such sharing and building capacity makes sense and should be facilitated by donor agencies. WHO, through its creation of a Department of Research Policy and Cooperation within the cluster of Evidence and Information for Policy, has defined as one of its objectives: "the development of initiatives aimed at strengthening research capacity in the developing world with the ultimate aim of enshrining research as a foundation for policy".

A number of other international initiatives have also attempted to address some of these capacity issues: the International Health Policy Program (IHPP), the Applied Research on Child Health (ARCH) project, the Swiss Commission for Research Partnership with Developing Countries (KAPE) and, in Canada, the IDRC. Since 1970, IDRC has been providing financial and technical assistance to academic institutions, government agencies and NGOs in developing countries, as a means of promoting sustainable and practical development and strengthening indigenous research capacity. IDRC's experience provides important and valuable lessons about implementing applied research in partnership with NGOs [30], as summarized in the table 1.

**Table 1:** Lessons learned from research in partnership with NGOs: IDRC experience

*First, applied research should have a practical application, reinforce knowledge and skills, and introduce and promote innovative, effective strategies and approaches for improving human health and well-being. Not only should research results be for local application, they should also be shared and adapted to other venues and contexts.*
*Second, efforts need to be made to build knowledge and understanding about the benefits accruing from applied research. NGOs, by their very nature, are action-oriented. Applied research is often perceived as of limited use to their ends, an esoteric, academic exercise of limited value to the immediate needs of the poor and disadvantaged. Time and effort need to be invested in nurturing an understanding within the academic community of the value of applied research within the context of development efforts.*
*Third, applied research should be used to develop and strengthen local research capabilities. NGOs do not, as a rule, possess the internal capacity and skills to design and conduct applied research studies. Attention should be paid to assisting NGOs in making contact with qualified researchers, and increasing NGO knowledge and skills to negotiate the terms of reference for applied research studies. This cannot be achieved simply through providing information about applied research methodologies or organizing a single workshop. Trust has to be developed between the NGO and academic communities, as a means of reinforcing linkages between them and building upon and using their comparative strengths, characteristics and areas of expertise to design and conduct applied research.*
*Fourth, local communities should be involved in the design and implementation of applied research activities. The local people need to understand the purpose of the proposed research, provide input and advice about its design and conduct, and be actively involved in the application and dissemination of research results. Without the active participation of the community, the utility and eventual application of the research results will be of little value.* *Source*: [30]

The CPHA, through the CIDA-funded initiative Canada's International Immunization Program – Phase 2 (CIIP2), dedicated 5% of the program's budget to applied research. Part of this funding was used to strengthen primary health care in developing countries through the NGOs that implemented the immunization and primary health care activities through the auspices of CIIP2.

NGOs who wish to become more involved in research generally recognize the need for extramural training and support. Partnering with universities and research institutions may provide such training opportunities. Additionally, there are international institutions such as INTRAC (International NGO Training and Research Centre) that are specifically geared towards meeting the challenges and needs of NGOs in research. Those NGOs that are part of international networks can draw from the body of research conducted elsewhere.

NGOs may also provide substantive input into research training, be it by grounding research methods in reality so that research is more applicable, or by providing research sites and questions for academia and graduate students. NGOs may also be in a good position to identify young scientists and promising investigators in host countries.

Stimulating the demand for research by user groups, rather than supply-driven research, is one of the three strategies identified by Harrison & Neufeld [31] for capacity-building for essential national health research. NGOs and communities as user groups could be the target of capacity-building efforts.

## 4 NGO involvement in health research

There is a lack of accessible and centralized information on NGO involvement in health research, although the CPHA CIIP2 applied research publication lists over 20 examples of NGO-related applied research carried out in the 1990s. The examples given below are based on discussions with a limited number of Canadian and international NGOs: CARE, World Vision Canada (WV), CECI (Centre d'étude et de coopération internationale), Inter Pares, HKI (Helen Keller International), and CCIC. In the case of AMREF (Africa Medical Research Foundation), ADI (Alzheimer's Disease International), Médecins sans Frontières (MSF) and RITC (Research for International Tobacco Control), most of the information was obtained from their websites and related publications and documents. The interviews and discussions covered the specifics of the implication of the NGO in health research, lessons learned through the experience, and respondents' perceptions on the role of NGOs in global health research, and on the strengths and weaknesses of their organization in this regard. These selected NGOs provide insight into some of the critical issues facing NGO involvement in global health research. It should be kept in mind that this selection is small and not meant to be representative. Nonetheless, all of these NGOs are involved, directly or indirectly, in global health research, and they are all Canadian or present in Canada.

### 4.1 NGOs and their involvement in global health research: illustration cases

The interviews covered a broad range of cases, from NGOs little involved in research to those actually conducting independent research. The types of involvement are briefly described below. A salient observation is that what is considered as research by different NGOs is, for the most part, unclear and highly variable. This suggests the need for NGOs to develop common views on what is research, the various types of research, and the components of the research process. The interviews also revealed that while some NGOs are reluctant to be involved in research, others are eager to strengthen their capacity to do so.

CECI has long been involved in health research, although it is reluctant to call this 'research'. A major activity is the undertaking of baseline studies that typically include an assessment of the health and nutritional status of populations. The data are used to orient or reorient programs, and to inform communities. In Cambodia, for instance, it conducted an initial assessment for a project aimed at improving the livelihood of rural poor in two sectors: health/nutrition, and agriculture marketing (CECI and Cambodia Researchers for Development: Improving Livelihood of the Cambodian Rural Poor: Strategies in Health, Nutrition and Agricultural Commodity Marketing, 2001). One interesting aspect of its recent work is the 'policy feedback' that it conducts in its large projects. The intent of the analysis is to clearly identify the lessons learned, and to discuss these with decision-makers and technical officers. This may be considered as part of 'knowledge translation' and it can be a particularly useful approach in advancing policies and programs. While CECI is also involved in health projects that do not include research even in a broad sense, it conducts research in areas that are indirectly related to health. For instance, in the IDRC-funded project intended to alleviate poverty in Burkina Faso, Viet Nam and Nepal, it collaborates with local universities and research institutions for the research and training components, notably on adapting the assessment of poverty to the specific context.

CCIC and its member NGOs are involved in international policy research. For instance, Trade-Related Aspects of Intellectual Property Rights (TRIPS) agreements have implications on access to drugs. In the reorientation of CIDA for improved aid effectiveness, there are obvious health implications, including how to respond to health plans as defined by health ministries, and assist with poverty reduction strategies. CCIC sees research on policies as a critical role of NGOs, and considers that NGOs should be more involved in the policy debate both in Canada and globally.

World Vision (WV) Canada is active in research, particularly (but not only) in the framework of its MICAH projects (Micronutrients and Health in Africa) funded by CIDA. It primarily conducts formative and evaluation research (see table 2 for report of findings in Sénégal, published jointly with CIDA). Although it has PhD or MSc level personnel in each of its technical units, it does not have in-house research expertise *per se*; it partners with research institutions, in the field and in Canada. It does not have the capacity to analyze all the data that it collects and therefore it collaborates with academic institutions in Canada. Graduate students can use the data for their theses. WV officers may also sit on graduate students' supervisory or examining committees. The primary use of the research findings is to reorient programs and inform the community. As programs may have to change their operations as a result of such research, the exercise may, at times, be regarded as threatening.

**Table 2:** Final evaluation report, World Vision Canada, Micronutrient-for-Health Project in Sénégal (2002)

*The objectives of the project initiated in 4 districts in 1997 were to reduce micronutrient malnutrition among women and children, to reduce the incidence of illnesses affecting micronutrient status, and to strengthen local capacity for controlling micronutrient malnutrition. The baseline study revealed a high rate of (iron deficiency) anemia in pregnancy (49%), of low retinol (vitamin A) levels in breastmilk (57%), and of low serum retinol concentrations among preschool-age children. Iodine deficiency was widespread, with 20% of school-age children showing severely low urinary iodine levels. A similar survey was conducted after 4 years of project activities, and included control zones in each district. The final evaluation showed an almost complete elimination of vitamin A deficiency in the project areas, which was primarily attributable to the high coverage of vitamin A supplementation of under-fives and postpartum women. Household use of iodized salt increased from 6% to 14%. Anemia remained high among pregnant women (44%), however, in spite of the iron-folate supplementation scheme. The rate of intestinal parasites declined, but the project did not have an impact on diarrhea. The MICAH project had a positive impact in strengthening the national vitamin A policy of Sénégal. The evaluation report was published by the project and widely disseminated. The survey findings and recommendations were fed into the design of an up-scaling phase of the project, with more emphasis on the reduction of anemia among women.*

CARE is directly involved in research, and its involvement covers the whole process from conceptualization of the research question to data management and dissemination of research results. Some offices have staff whose role is specifically research-related, but this varies. They also work with partners. CARE has even been contracted by some donors to conduct research. The research is primarily qualitative, including participatory approaches, as well as operations and action research. CARE also conducts surveys, situation analyses and policy reviews. It receives funding for research from bilateral and multilateral agencies, and from large organizations such as Family Health International and the Population Council.

Helen Keller International (HKI) is a technical assistance NGO that is also directly involved in research as part of its mandate. It addresses the causes of preventable blindness. It also provides rehabilitation services to blind people, and helps reduce micronutrient malnutrition which can cause blindness and death in children. It is involved in most stages of the research cycle, focusing on operations and action research. HKI's focus on blindness and micronutrients is a strength in that its research is more focused than that of other NGOs involved in health and nutrition. Its funds for research come from different sources. A research component may be built into programs, some operational research is conducted with funds for surveillance, or funds are provided for R&D specifically (eg. for FRAT studies [Fortification Rapid Assessment Technique]) and for the development of tools to assess the quality of nutrition interventions leading to adoption of relevant strategies (in Mozambique, Burkina, Mali and Niger). HKI has in-house expertise in research. There are several full-time research positions. In addition, it works with research partners at the local level, as well as with universities in Canada and USA.

Inter Pares was created in 1975 to support NGOs from the South and to provide international development education in Canada. Inter Pares uses its own funds to conduct social research on political and economic issues, primarily action research. For instance, it carried out collaborative research with NGOs in the Philippines and of Bangladesh on family planning policy, and in Africa it has carried out research on economic issues. With *Forum Afrique Canada *, for instance, it is studying Canadian government trade and aid policy after G-8. It has in-house research expertise, particularly in sociology, although there is no research position as such. It usually works with partners, as it is a small NGO. Inter Pares uses research findings mainly for education and advocacy.

AMREF has been active since 1957 in the field of applied health research and has an extensive bibliography documenting research results in the form of peer-reviewed publications, theses, manuals, reports, abstracts and conference presentations. The focus of AMREF's research activities has been primarily in the operational and applied domains. Many have addressed the important disease burden caused by communicable diseases such as malaria (see table 3) and schistosomiasis, but others have addressed organizational issues such as health information systems and technological issues like field diagnostics.

**Table 3:** Example of an AMREF research study listed in its extensive bibliography

*In 1995, D'Allessandro et al *[32]*published a study which compared the efficacy of insecticide-treated and untreated bednets in preventing malaria in children living in the Gambia. The survey included 2300 children between the ages of 1 and 4 years; 1500 from villages who had received insecticide-treated bednets within their primary health care and 800 from villages which had not received treated bednets. It was found that the greatest benefit, in terms of reduced malaria morbidity, was observed in children who slept regularly under treated bednets. Measurable benefits were also accrued in children who slept regularly under untreated bednets, compared to children who did not use bednets at all. The conclusion of this study was that educational campaigns might well promote even the use of untreated nets because of the additional health benefits, while ultimately aiming at coverage with insecticide-treated bednets.*

Alzheimer 's Disease International (ADI), an NGO affiliated with WHO, specifically provides support for research among its numerous activities. In particular, it supports the research work of the 10/66 Dementia Research Group (the 10/66 refers to the dementia research gap, in which 'less than one-tenth of all population-based research into dementia is directed towards the two-thirds or more of cases living in developing parts of the world [31]). The vision of ADI is that research not only generates awareness, but is the basis for policy which, subsequently, can provide the impetus for development of appropriate services for affected persons. The 10/66 Dementia Research Group divides its research activities into pilot studies, qualitative studies, intervention studies and population-based studies. This group has published a consensus statement [33] and a methods paper [34], and members are now publishing research results (see table 4). This NGO's 10/66 Dementia Research Group has regional networks in India and South Asia, Latin America and the Caribbean, China and South East Asia, Africa and Russia, Eastern and South Eastern Europe which are coordinated by Dr. Martin Prince of the Institute of Psychiatry in London, England.

**Table 4:** Example of an Alzheimer's Disease International- supported health research study

*The results of a population-based study undertaken in Kerala, India to evaluate a community dementia case-finding program was published by Shaji and collaborators in 2002 *[35]. *Their aim had been to validate a training program where local community health workers (CHWs) were trained to identify possible cases of dementia. The training program consisted of 2 ½ hours of formal instruction. Workers' diagnoses were then confirmed by an experienced psychiatrist. The 19 CHWs identified 51 possible cases among 1979 persons aged 60 and older in their communities. There was expert confirmation for 33 of these cases (65%). Although the remaining 18 did not have dementia, 13 did in fact suffer from other psychiatric illnesses and only 5 had no psychiatric diagnosis at all. The conclusion was that CHWs can play an important role in identifying cases of dementia in a community setting.*

Médecins sans Frontières (MSF) was the first NGO to both provide emergency medical assistance and publicly bear witness to the plight of the populations they served. MSF is at the forefront of emergency health care as well as care for populations suffering from endemic diseases and neglect. MSF has undertaken an initiative on drugs for neglected infectious disease which combines advocacy, research and capacity development, and networking. In contrast with private sector research, it is need-driven rather than profit-driven. Five pilot projects are currently underway focusing on capacity building and technology transfer. This initiative started with a review of pharmaceutical research and development outcomes over the last 25 years and of current private and public initiatives. Highlights of the findings and conclusions, published in Lancet [36], are provided in table 5.

**Table 5:** Analysis of trends, drug research and development for tropical diseases, MSF (2002)

*The extensive review revealed that of 1393 new chemical entities marketed between 1975 and 1999, only 16 were for tropical diseases and tuberculosis. All new drugs for neglected diseases represented a clear therapeutic benefit, and all are included in the WHO Essential Drug List, which indicates the importance of new drugs for neglected diseases. In contrast, over the same period, two out of three new drugs offered little advantage over existing ones. There was no indication that drug development for neglected diseases would significantly improve in the near future, however. Private-public partnerships, or else, incentives to encourage private investment towards the development of new cost-effective drugs may help overcome this limitation. For the most neglected tropical diseases which may not account for a large share of the global burden of disease, a new approach is needed. The feasibility of an international not-for-profit network that would focus on the most neglected diseases is being tested in the on-going pilot projects.*

Research for International Tobacco Control (RITC) is an International Secretariat based at IDRC headquarters (Ottawa) that funds multidisciplinary tobacco control research projects in developing countries. Its mission is to create a strong research, funding and knowledge base for the development of effective tobacco control policies and programs, through a combination of research, dissemination, strengthening of capacity and coordination. RITC concentrates on research on psycho-social correlates of tobacco use. It provides support to research projects conducted by NGOs, such as, the Youth and Tobacco Survey conducted in Russia by the Russian Public Health Association, with the technical assistance of the CPHA (table 6).

**Table 6:** Youth and Tobacco Survey, Russian Public Health Association (RPHA), 1999

*This study is part of the Global Youth and Tobacco Survey (GYTS) undertaken in several countries around the world. The survey in Russia was designed to provide prevalence data on tobacco use among adolescents in school (13–16 years), and to better understand and assess students' knowledge, attitudes and practices related to tobacco. Information pertained to, for instance, age of initiation of tobacco use, perceived health risks and social benefits, extent of peer and advertising pressure, perception of the tobacco-related curriculum, and likelihood that tobacco users will quit. The survey raised awareness on the issue of smoking and youth. Several recommendations were made by the Association to Parliament on the basis of survey findings, including the adoption of legislation to limit tobacco advertising, to reduce the tar and nicotine content of cigarettes, and to have an impressive warning labeling on packages. Seminars and conferences on the survey results and their implications were held. The Association has prepared a report: "Tobacco or Health in Russia". Additionally, the President of the RPHA, as a result of the GYTS survey and the ground-breaking leadership role the RPHA played in tobacco control among youth in Russia, was a member of the delegation from the Russian Federation to the WHO Framework Convention on Tobacco Control (FCTC) – hence an example of the translation of applied research results into policy action.*

CPHA has also provided technical and financial support through its various international initiatives funded by CIDA to its public health association partners to carry out of the GYTS in Burkina Faso, Niger, Haiti, and Cuba; and in partnership with Institutes of Public Health in Bosnia & Herzegovina, Serbia & Montenegro, and in the UN-administered province of Kosovo. The results from the surveys (carried out in collaboration with the Centers for Disease Control and Prevention [CDC] of USA) are being used to develop tobacco control policy and youth-focused smoking prevention and cessation programs.

The experience of CSIH in global health research is described under 5.2.1.

### 4.2 NGOs' perceived strengths and weaknesses in research

The following summary of perceived NGO strengths (and weaknesses) in health research is based primarily on the data from individual and group interviews that were specifically conducted as inputs to this discussion paper.

It is a common view that NGOs are in a good position to participate in health research because of their knowledge of, and their presence in, local communities. Furthermore, their involvement increases the relevance of research to communities. "*NGOs give a human face to research, and they are in a good position also to build on indigenous capacity*" (Interview with S. Baker, HKI Africa). Additionally, they may be more compelled to complete the research cycle and apply the findings. Their involvement in research is perceived as a motivation to use the research to design, develop and respond to circumstances affecting development. Evaluation research, in which they are frequently involved, tells them whether or not they have an impact.

Since they are closely connected with communities, they have the ability to see the application of their research results. For this reason, NGO-initiated research is often more likely to be translated into practice in a timely manner as it is almost always directly related to practice. The NGO structure brings concreteness and a style which is guided by values and beliefs with an action orientation.

Another major strength of some NGOs is their international networks which give them access to technical information and support. Finally, NGO values of ethics, solidarity and dialogue are important for health research to contribute to reducing inequities and for empowerment.

### 4.3 Constraints to greater NGO involvement in health research

#### 4.3.1 NGO views on research and its congruence with their mandate

Many reasons that account for the reluctance of NGOs to become (more) involved in research activities pertain to NGO perceptions on research (Center for Advanced Studies of International Development. Symposium on NGO/Academic Linkages. East Lansing: Michigan State University; April 16–17, 1993; Edwards M, Griffiths M: Terms of Reference for the DSA [Development Studies Association] Workshop on the Academic Practitioner Interface. London: DSA, 1994). In the past, research was an academia-driven and based, elitist and theoretical exercise, the results of which are of little use to NGOs and the communities with which they work. Traditional research strategies and approaches were seen as top-down, non-participative and controlled by external actors. Research activities were regarded as requiring special technical expertise, much time and effort, access to professional journals and research literature, and substantial human and financial resources, characteristics not typically found in NGOs. Finally, the scientific rigor demanded by researchers was believed to be difficult to achieve in field-based situations, where unpredictability and subjectivity are the norm.

What NGOs perceive as research and as their role in this respect varies widely. For instance, there is some hesitation and even reluctance in including baseline studies or project evaluation under 'research'.

Another obstacle is the fact that some NGOs that raise funds from the public are afraid to go against the expectations of the donors if the money is reallocated for research, and particularly for policy research in Canada: "*Donors do not want to hear that NGOs are doing research as they are implementation organizations*" (Interview with C. MacDonald, World Vision Canada).

#### 4.3.2 Lack of training opportunities, funding, time and motivation

Among other barriers, interview respondents mentioned lack of training opportunities, lack of funding owing to their (limited) mandate, priorities of funding agencies, and time constraints.

Because of lack of training or of specialized researchers, NGOs may not be in a position to conduct top quality research, and scientific rigor may be lacking in certain instances. Lack of access to scientific literature when in the field can also be a major shortfall.

It is often difficult to secure research funding from certain donors. Many Canadian NGOs rely heavily on negotiated contracts with CIDA, which leaves little time and place for research. However, CIDA does fund some research (in a broad sense), particularly formative and summative evaluations. These are encouraging trends, but the aim should be for bilateral agencies to openly fund some research, like in the UK and some Scandinavian countries.

Lack of interest or of a clear view of the whole research process can also be considered as impediments. As several NGOs do not see research as part of their mandate, they may not be willing to get involved in research: "*NGOs do not have a research mandate, and therefore we do not foresee developing research expertise in-house. Linking for instance with universities is feasible for development-driven research*" (Interview with R. Hazel, CECI). NGOs may have to change their structures and priorities in order to support autonomous research.

#### 4.3.3 Scale and type of NGO research

Because NGO research is often conducted on a small scale and is usually of a qualitative nature, it often goes unrecognized by governments, and even by research organizations and funding agencies, which tend to favour large scale quantitative research.

NGOs interested in pursuing a research profile require a type of mentorship in terms of standard performance indicators in the research domain. For example, publication has traditionally not been a strength and much NGO research does not reach beyond the gray literature or report level. Because scientific publications are an important means of transfer or dissemination of research results, NGO capacity to publish their findings needs to be strengthened.

#### 4.3.4 Weak links with the international research community

There is not enough networking and collaboration between NGOs and the international research community, including academia. This has traditionally been due to a dichotomy in the interests of NGOs and the academic community, in that NGOs are more oriented towards a development agenda, while academics tend towards special research interests.

## 5 Future needs

In light of the above issues and concerns, and in order to foster greater interest and participation of NGOs in research, the barriers of lack of interest, lack of funds, lack of training and lack of recognition, among others, need to be addressed. We discuss some strategies below.

### 5.1 Opportunities to build NGO capacity in research, in Canada and overseas

Substantial global health academic capacity has developed within NGOs both in the U.S. and the U.K. For example, Family Health International (FHI) has integrated research, training, and development capacity on an evidence-based foundation. It also embodies many of the competitive aspects of private sector-led initiatives that can allow creativity and innovation.

As emphasized by Harrison and Neufeld [31], however, capacity building efforts for health research have been of most benefit to industrialized countries. In order to ensure that less developed countries are the principal beneficiaries, they recommend, as part of a three-pronged strategy, the nurturing and support of multi-stakeholder problem-oriented learning, and research networks which include NGOs. The other components of the strategy are research investments that explicitly reduce the high cost of knowledge translation in developing countries, and the stimulation of demand-driven research.

A peer-learning process is among the strategies for NGO capacity building in research. NGOs can draw on expertise already developed in research-based NGOs. The learning process should be shared with NGOs from the North and from the South. Additionally, NGOs should consider taking the initiative in organizing scientific activities (seminars, workshops, symposia) on global health research topics, which could serve as a catalyst in bringing together different stakeholders.

### 5.2 Building partnerships and alliances

#### 5.2.1 Creating and facilitating networks that support global health research

The creation of networks which have the common goal of supporting global health research is one way to strengthen partnerships and to consolidate valuable resources from each partner. Leadership and governance issues are necessary hurdles which can be overcome by focusing on the ultimate gains in terms of supporting and conducting successful research activities. NGOs can assist in the establishment and functioning of these networks, particularly by providing stable infrastructure support. One of the greatest challenges is in making the network function effectively through different leadership turnovers in the different partner organizations.

CSIH has had global health research as part of its mandate since its formation. CSIH is an active member of the Canadian Coalition for Global Health Research (CCGHR), and a key challenge in this capacity is to develop a strong foundation of understanding and mutual respect amongst all players in global health research, including NGOs. CSIH experience in global health research is described in table 7.

**Table 7:** The experience of CSIH in global health research

*In the early years, CSIH partnered with the Canadian University Consortium for Health In Development, which represented all the major universities in Canada and their partners in research and development. This partnership represented a strong and vital part of CSIH's operations. Following the decrease in funding for such a partnership, there was a decision to disband the Consortium and establish a network of universities and colleges that would promote and support academic and research interests within the Society. This network, which was formally given the status of a Division for a few years, has been and continues to be functional but not as a strong advocacy unit. This was largely due to the fact that funding for the network was cut by CIDA in 2000. Nevertheless the network is an important source of technical support for CSIH in its projects and advice.*
*Following the Thailand meeting in October 2000, Canadians were challenged to explore the role that they could play in diminishing the 10/90 Gap in Global Health Research funding available to Low and Middle Income Countries (LMIC). To this end an interest group was formed, of which CSIH was part in order to carry on the momentum of Thailand and future explorations.*
*Key people met with decision makers during the spring and summer of 2000 and September 11, 2001, marked the inaugural workshop in Vancouver to discuss global health research and the 10/90 Gap. CSIH was one of two NGOs who attended. Following that meeting, CSIH was invited to participate in a new Canadian Coalition for Global Health Research (CCGHR). CSIH was active in suggesting that the concept of a coalition was a way to emphasize the role of advocacy and action that is necessary for global health research initiatives to be successful.*
*As of October 2001, the Coalition included two NGOs (CSIH and CPHA) who were part of a lobby to expand the mandate of CIHR (Canadian Institutes of Health Research) to include global health research in more than one Institute. To this end, the Institutes of Gender and of Aboriginal Health joined the Institute of Population Health in realizing its global health mandate.*
*The Global Health Research Initiative memorandum of understanding (MOU) was signed at the 2001 Canadian Conference for International Health (hosted by CSIH). The amendment to MOU as a result of negotiations between CSIH and CIHR included NGOs as one of the important players.*
*The first formal retreat for the coalition was held in August 2002. CSIH was formally named as a member of the Coalition Steering Group. The Working Group on the Role of NGOs in Research was affirmed as separate from the Advocacy Working Group. CSIH agreed to take the lead to collaborate with other key NGOs to develop a paper and case studies.*
*CSIH as part of CCGHR lobbied in the spring and summer of 2002 to the G-8 for the inclusion of a commitment to global health research within NEPAD (New Partnership for Africa's Development). Support for global health research in Africa was announced and funds were set aside for this new initiative.*
*The first Annual Meeting of CCGHR was held at the Canadian Conference on International Health (CCIH) in October 2002. The Working Groups reported at that meeting and CSIH announced the formation of a Research Committee and invited its members to participate. The Executive Director drafted an outline of a background paper on the Role of NGOs in Global Health Research and presented to the plenary session of the annual CCGHR meeting for comment and feed-back. An ad hoc Working Group on Research was formed to draft the background paper with a view that it will be a position paper for CSIH and provide a background working paper for the Coalition.*
*In the autumn of 2002, the first request for proposals for global health research grants was released. Despite the fact that NGOs were named as important partners, they were not invited to be part of the review panel for this round. It was noted as a deficiency in the review of the process by CIHR.*
*To date, CSIH has been an active and welcomed participant of all key meeting of CCGHR Steering Committee meetings. CSIH remains actively engaged in working groups on Governance to determine options for institutionalization of the CCGHR. In collaboration with CIHR and IDRC, CSIH is actively planning the Second Annual Global Health Research Meetings at CCIH and the integration of significant research and development content in the conference.*

#### 5.2.2 Partnerships with universities and other research institutions

Partnerships with universities and other research institutions is one means of strengthening the research capacity of NGOs, and also of academia. NGOs and research organizations each have a unique 'value-added' contribution to make to global health research and therefore, partnering among them amplifies their individual strengths. Such partnering may be a real challenge for NGOs, however, as their institutional culture is so different. NGOs may be invited by universities to partner, but plans are often already laid out, so that the NGOs may only be involved in executing the plans. What NGOs want is to be part of the research process from the start. An interesting initiative to document and promote South-Canada health research partnerships is currently underway [37].

NGOs are open to partnerships with academia, but the goal has to be development-oriented. Experience suggests that it is often difficult to reconcile the academic and development framework, for instance when integrating MSc or PhD students in development projects. Nonetheless, integrating graduate students in NGO projects should be a good strategy for balanced and equal partnerships.

As less than 1% of university-based health research in Canada is directed towards the problems of global health according to a Canadian Consultation on Global Health Research held in 2001 , the prospects of university-NGO research links are constrained by funding. Nevertheless, the recent Global Health Research Initiative of the coalition of Canadian institutions funding global health research is promising as it opens new avenues for research collaboration between the North and the South, and hopefully also between universities and NGOs.

During the 1990s, there were several attempts to bridge the NGO/academic gap with respect to health research in developing countries. Save the Children UK, Oxfam and some US-based institutions supported workshops and symposia that aimed at bringing together representatives from both communities as a means of building links and forging partnerships in support of increasing the scale, scope and relevancy of health research in developing countries (Edwards M, Griffiths M: Terms of Reference for the DSA [Development Studies Association] Workshop on the Academic Practitioner Interface. London: DSA, 1994). CPHA, through the CIDA-funded Canada's International Immunization Program – Phase 2 (CIIP2), supported applied research carried out by Laval University, Université de Montreal and University of British Columbia. At the time, these were quite innovative approaches to applied health research, linking the universities with local NGOs. In 1995 CPHA also organized a Symposium on NGO/University Linkages for Health Research [30].

One mechanism to expand the use of research generated by NGOs is to improve the linkage between NGOs and universities. Each complements the other in the area of health research. NGOs offer proximity to people and situations, reality-based and context-specific research environments, opportunities to develop and assess innovative strategies and research methods, a means of disseminating and popularizing the results of research projects, and credibility outside of academia. Universities and other research organizations offer expertise in research design and application, an environment for reflection, access to and knowledge about most recent literature, a tradition of scientific rigor and interest in new, innovative research methods and approaches, and a high degree of credibility. Academics can also provide guidance and advice on how to prepare research proposals and to carry out research studies, guidance in the preparation of reports and publishing of research results, and training for NGO staff in research methods.

The participants of the CPHA Symposium on NGO/University Linkages for Health Research in Developing Countries [30] identified several mechanisms that could help bridge the gap between NGOs and universities as a means of facilitating future collaborative research initiatives. There must be first and foremost a real willingness on the part of both parties to modify their attitudes about the role and capabilities that each can offer. Mechanisms to achieve this end include conferences and seminars, newsletters, and the use of e-mail and the Internet. Another suggestion called for the use of "field-friendly" research methodologies. It was also recommended that, although the objective is not to transform NGOs into research institutes, they should receive more training in research methods and proposal development. It was suggested that exchanges take place wherein university researchers use sabbatical leave to work with NGOs and NGO personnel be seconded to universities to provide a field perspective. Additionally, research results need to be disseminated quickly and in a format that ensures maximum access by those in the field who are to apply the knowledge generated. Otherwise, research creates expectations within the NGO community and study population that remain unsatisfied.

The development of innovative North-South research partnerships is the focus of a working paper prepared for the CCGHR by Neufeld et al [37]. As emphasized in this document, such partnerships are not an end in themselves, but rather, they are to contribute to sustainable health research systems and to health development. Principles of research partnerships, and a useful model to assess these, are proposed. Although types of partnerships are not specifically detailed, it is implied that NGOs are important research partners.

Finally, as mentioned earlier, stronger partnerships between NGOs and social science researchers in particular should be sought in resource-poor countries. Lessons learned from these partnerships, in the areas of action and indeed policy and legislation (for example, in the tobacco or environment fields) show how evidence can be transformed into action with the right partnerships between researchers and NGOs.

#### 5.2.3 An NGO Network for Global Health Research?

NGOs may benefit in various ways from developing a global health research network. First, many NGOs already operate at national and international levels and understand the challenges of coordination and communication which this entails. There is a need to identify overarching principles of NGO contribution to health research. Second, NGOs must be both proactive and interactive within the framework of the health research agenda. Roles must be clearly understood by each partner. In any case, advocacy for relevant research and use of results would be a critical function of the network (see training modules on advocacy [38]). Such networks may enhance the ability of NGOs to partner with other research stakeholders in multisectoral coalitions, and even to initiate partnerships with research organizations.

In the framework of an NGO network of this sort, the following discrete activities could be envisaged by the lead NGO:

• To invite NGOs to post on a selected website success stories, as well as their experience/opinions/needs/priority research issues, using a template adapted from the one developed for this purpose in the UK

• To organize workshops for NGOs who are, or who wish to become, involved in research, with research organizations where deemed appropriate.

The purpose would be:

• To link these NGOs in order for them to interact on research issues;

• To share lessons learned and success stories of research involvement of NGOs and their partners

• To enhance understanding of, and collaboration with, potential research partners;

• To set-up a core group of NGOs involved in global health research to convey NGO views to global health research fora and organizations.

The following are a few key questions that could be addressed by an NGO network:

1. How can NGOs contribute to the framing of the research questions if we were to support the necessary equity-based research for improving the overall performance of the health system?

2. How do we balance this with the necessity for research which documents and monitors sustained and emerging inequities which may have a greater impact on the health and well being of individuals than the health (care) system will ever have?

3. How can we ensure that NGOs influence research priorities so that they are reflective and evaluative of overseas development assistance (ODA) direction and priorities such as national poverty reduction strategies. For example what is the impact of PRSPs on equity and health? How will researchers monitor this? What could be the potential role of NGOs in partnership with researchers to begin to monitor and evaluate this new direction in overall aid policy?

4. How can NGOs be best represented within the international research community?

The new millennium offers many challenges. In order to maximize the potential benefits of health research, all partners including NGOs must share a common vision and recognize and appreciate the strengths of each. Participation in health research needs to be a coordinated effort. One key challenge will be to establish better communication among all partners in health research. This can only be achieved by a willingness to share in leadership, ownership and in the conduct of health research activities. Another key challenge will be to explore ways in which funding for health research can be strengthened. Leveraging must be seen as a strategic tool of NGOs to maximize dollars allocated to health research.

## Conclusion

Several NGOs have had impressive track records in global health research. Other NGOs have expressed an interest in becoming more involved in global health research. Their contribution to more equitable, ethical, relevant and effective research is crucial and needs to be strengthened. Research has to be regarded as a broad loop system rather than restricted narrowly to the production of knowledge. This is particularly critical for global health research whose primary goal should be to improve health and its determinants in low and middle income countries. NGOs principal roles in the process pertain to shaping research priorities, advocacy for more relevant research, translating and using research findings, in addition to generating new knowledge in areas where they may have a comparative advantage, notably qualitative, social, action, evaluative, and policy research. NGO partnerships with research organizations should be seen as means of a mutual enhancement of health research capacity and contribution to development. NGOs should be instrumental in building with other stakeholders coalitions for global health research with the aim of closing the 10/90 health research gap.

## List of Abbreviations

ADI Alzheimer's Disease International



AFRO-NETS African Networks for Health Research and Development



AIDS Acquired immunodeficiency syndrome

AMREF African Medical and Research Foundation



ARCH Applied Research on Child Health (ARCH) Project



CCIC Canadian Council for International Cooperation



CCISD Centre de coopération internationale en santé et développement



CCIH Canadian Conference on International Health (hosted by CSIH)

CECI Centre canadien d'étude et de coopération internationale



CCGHR Canadian Coalition for Global Health Research



CDC Centers for Disease Control and Prevention



CHW Community health worker

CIDA Canadian International Development Agency



CIHR Canadian Institutes of Health Research



CIIP2 Canada's International Immunization Program – Phase 2

COHRED Council on Health Research for Development



CPHA Canadian Public Health Association



CSIH Canadian Society for International Health



DFID Department for International Development (UK)



DSA Development Studies Association



ENHR Essential National Health Research

FCTC Framework Convention on Tobacco Control (WHO)



FHI Family Health International



FRAT Fortification Rapid Assessment Technique

GYTS Global Youth and Tobacco Survey (WHO and CDC)

HKI Helen Keller International



IDRC International Development Research Centre



IHPP International Health Policy Program

INTRAC International NGO Training and Research Centre



KFPE Commission for Research Partnerships with Developing Countries



LMIC Low and middle-income countries

MICAH Micronutrients and Health in Africa (WV project)

MSF Médecins sans frontières



NEPAD New Partnership for Africa's Development



NGO Non-governmental organization

OECD Organization for Economic Co-Operation and Development



ODA Overseas Development Assistance

PRSP Poverty Reduction Strategy Papers

RITC Research for International Tobacco Control



RPHA Russian Public Health Association



SHARED Scientists for Health and Research for Development



TRIPS Trade-Related Aspects of Intellectual Property Rights

UNDP United Nations Development Programme



WHO World Health Organization



WV World Vision (Canada)



## Authors' contributions

HD designed the outline of the paper, conducted interviews with NGO representatives, wrote the first complete draft, and coordinated the review process within the CSIH. JHR drafted some sections and provided comments on the successive versions of the papers. MM provided international NGO and a field based perspectives to the paper, in addition to conducting group discussions with NGO personnel. LJ designed the figures, and edited and formatted the text. TG contributed to the conceptualisation and content of the manuscript. She wrote several sections and edited others, and she provided case studies.
